# Psyllium Improves the Quality and Shelf Life of Gluten-Free Bread

**DOI:** 10.3390/foods10050954

**Published:** 2021-04-27

**Authors:** Camilly Fratelli, Fernanda Garcia Santos, Denise Garcia Muniz, Sascha Habu, Anna Rafaela Cavalcante Braga, Vanessa Dias Capriles

**Affiliations:** 1 Department of Biosciences, Institute of Health and Society (Campus Baixada Santista), Federal University of São Paulo, Rua Silva Jardim, 136, Santos CEP 11015-020, Brazil; fratelli.camilly@unifesp.br (C.F.); fg.santos@unifesp.br (F.G.S.); de.garcia.nutri@gmail.com (D.G.M.); habu@utfpr.edu.br (S.H.); anna.braga@unifesp.br (A.R.C.B.); 2 Department Research, Pro Rectory of Research and Post-Graduation, Federal University of Technology—Paraná, Av. Silva Jardim, 775, Curitiba CEP 85504-311, Brazil; 3 Department of Chemical Engineering, Campus Diadema, Federal University of São Paulo, Rua São Nicolau 201, Diadema CEP 09972-270, Brazil

**Keywords:** bread quality, gluten-free, acceptability, multiple factor analysis, staling process

## Abstract

Psyllium husk powder was investigated for its ability to improve the quality and shelf life of gluten-free bread. Gluten-free bread formulations containing 2.86%, 7.14%, and 17.14% psyllium by flour weight basis were compared to the control gluten-free bread and wheat bread in terms of performance. The effect of time on crumb moisture and firmness, microbial safety, and sensory acceptability using a 10-cm scale was assessed at 0, 24, 48, and 72 h postproduction. Crumb firming was observed during the storage time, especially for the control gluten-free bread, which had a crumb firmness 8-fold higher than that of the wheat bread. Psyllium addition decreased the crumb firmness values by 65–75% compared to those of the control gluten-free bread during 72 h of storage. The longest delay in bread staling was observed with a 17.14% psyllium addition. The psyllium-enriched gluten-free bread was well accepted during 72 h of storage, and the acceptability scores for aroma, texture, and flavor ranged from 6.8 to 8.3, which resembled those of wheat bread. The results showed that the addition of 17.14% psyllium to the formulation improved the structure, appearance, texture, and acceptability of gluten-free bread and delayed bread staling, resembling physical and sensory properties of wheat bread samples during 72 h of storage. Therefore, according to the obtained results, this approach seems to be promising to overcome some of the limitations of gluten-free breadmaking.

## 1. Introduction

The key role of wheat gluten in breadmaking and bread quality cannot be replaced by a single ingredient. Hence, gluten-free (GF) breadmaking basically removes the most crucial ingredient for bread structure and quality. The lack of gluten has a high influence on dough properties, the breadmaking process, and the final quality and shelf life of gluten-free bread (GFB) [1,2]. As a result, obtaining high-quality GFB remains a major challenge for food scientists and producers, with increasing demand due to the growing number of individuals following a GF diet [2].

There has been a rise in the number of people adhering to the GF diet partly due to an increased prevalence and awareness of gluten-related disorders, especially celiac disease, which has become a notorious public health problem worldwide but mainly due to the widespread belief that a GF diet is healthier and more suitable for weight management [3,4,5]. Thus, this increasing demand and consumption of GF products is becoming a trend in the global food sector [2].

However, GFBs are characterized by a shorter shelf life than wheat bread, owing not only to starch retrogradation and the migration of water from the crumb to the crust but also (and especially) to the starchy raw materials and high water content used, two factors that accelerate staling and increase susceptibility to microbially driven deterioration [5]. Thus, constant efforts by researchers, as previously reported [1,6], are focused on the use of ingredients, additives, and technological alternatives to improve the quality of GFBs. The poorer quality of the GFBs and bakery products that are currently available on market is mainly due to the absence of gluten, which is able to guarantee optimal characteristics in wheat bread and bakery products [7,8]. This lack of gluten has a large negative impact on dough rheology and bread characteristics [9]. Gluten replacements are still the major challenge in the development of doughs and GF baked goods.

Recent literature reviews, such as those of Cappelli et al. (2020) [8] and Capriles et al. (2020) [2], pointed out various improvement strategies. The most active approach seeks to identify alternative ingredients that can mimic the viscoelastic properties of the gluten network, notably hydrocolloids, enzymes, and emulsifiers. Furthermore, another interesting improvement strategy to ameliorate GF dough and bread is based on the use of alternative proteins sources such as eggs, dairy products, and insects [10,11]) and pulses [12]), with additional benefits for the environment [13]. However, one of the most interesting improvement strategies for GF dough and bread is based on soluble dietary fiber application, of which psyllium husk powder is particularly rich, thus motivating this paper.

Therefore, the use of soluble dietary fibers has been suggested to improve the physical properties, sensory acceptance, shelf life, nutritional content, and glycemic response of GFB [14,15]. Among them, psyllium, a natural bioactive soluble fiber characterized as hydrocolloid extracted from the husks of *Plantago ovata* seeds, can improve health, aiding in intestinal transit, cholesterol control, satiety [16], and glycemia [17].

In addition to the health benefits, psyllium (P) presents water-binding, gelling, and structure-building properties that can increase the dough viscosity, strengthen the boundaries of the expanding cells, increase gas retention during baking, and improve the volume, in addition to reducing the loss of crumb moisture, softness, cohesiveness, and springiness during storage, thus improving the structure, texture, appearance, acceptance, and shelf life of the GFB [17,18,19,20,21], along with fiber enrichment, which decreases the glycemic index [17].

Among the studies cited, the one by Fratelli et al. (2018) [17] was the one that included the greatest P levels in GFB. These authors applied a factorial design to define the P and water (W) addition levels in GFB formulations to obtain a fiber-enriched and acceptable product. The optimum formulation was prepared with 2.86% P and 82.14% W flour weight basis (fwb), and various combinations of up to 17.14% P and 117.86% W levels could be applied to obtain acceptable GFBs. In comparison to the control, the P-added GFB had a 1.6- to 4-fold increase in the dietary fiber content. In addition, P was recognized as a promising ingredient to improve the physical, sensory [22], and nutritional properties of GFB by Fratelli et al. (2018) [17].

Based on previous findings, the present study aimed to investigate the ability of P to improve the quality and shelf life of GFB. Physicochemical, microbiological, and sensory analyses were performed to compare the P-added GFB formulations to the control GFB and wheat bread (WB) during 72 h of storage.

## 2. Material and Methods

### 2.1. Ingredients

The psyllium husk powder (VITACEL^®^ Psyllium P95) was supplied by JRS Latinoamericana Ltd. (São Paulo, Brazil). This is a dietary fiber concentrate from milled psyllium husks with 95% purity, an average particle size of 250 µm, and an approximately 80% dietary fiber content, as previously described by Fratelli et al. (2018) [17]. The other ingredients used in the breadmaking process were obtained from a local market.

The ingredients purchased in the local market (São Paulo, Brazil) used in the preparation of GFBs consisted of rice flour (Urbano Agroindustrial Ltd., Jaraguá do Sul, Brazil), cassava starch (General Mills Brasil Alimentos Ltd., São Paulo, Brazil), calcium propionate (Pantec Aditivos e Ingredientes para a Indústria, São Paulo, Brazil), bread spray mold inhibitors (Conserv, TFF Alimentos, São Paulo, Brazil), and water, eggs, sugar, soybean oil, salt, and dry yeast.

The WBs were prepared with commercial wheat flour (J. Macedo SA, São Paulo, Brazil), which had contents of ash 0.5%, fat 1.6%, protein 12.3%, total dietary fiber 3.2%, and available carbohydrates 82.4%. The wheat flour had 23% wet gluten according to the manufacturer’s instructions.

### 2.2. Methods

#### Preparation and Storage of Gluten-Free Breads

The GFBs were selected based on a previous study by Fratelli et al. (2018) [17] and were prepared with the following combinations: 0.00% P and 100.00% W; 2.86% P and 82.14% W; 7.14% P and 91.10% W; and 17.14% P and 117.86% W fwb (samples named GFB0P, GFB2.86P, GFB7.14P, and GFB17.14P, respectively).

The GFBs were prepared according to the procedure described by Fratelli et al. (2018) [17]. Briefly, the GFBs were prepared with the following formulation (g/100 g fwb): rice flour 75, cassava starch 25, whole egg 25, whole milk powder 10.5, white cane sugar 6, soy oil 6, salt 2, dry yeast 0.8, calcium propionate 0.1, and different combinations of P and W. The breads were prepared by the straight dough method, consisting of mixing the ingredients for 7 min at 110 rpm. The resulting doughs for GFB0P and GFP2.86P (400 g) were directly scraped into baking tins (19 × 7.5 × 5 cm³). Doughs of GFB7.14P and GFB17.14P were molded and then placed into tins. After 90 min of proofing at 40 °C and 85% relative humidity, the breads were baked at 140 °C for 45 min and cooled for 2 h.

Two WB formulations were prepared as described by Santos et al. (2018) [22]. The first wheat flour-based formulation (WB1) was prepared according to AACC standard method 10–10.03 [23]. The second formulation (WB2) was laboratory developed by adapting the ingredient proportions used in the GFB formulation.

The WBs were prepared as previously described by Santos et al. (2018) [22]. WB1 was prepared with the following formulation (g/100 g fwb): wheat flour 100, water 50, white cane sugar 6, soybean oil 3, salt 1.5, dried yeast 1.2, and calcium propionate 0.1. WB2 was prepared with (g/100 g fwb): wheat flour 100, water 33, whole egg 25, whole milk powder 10.5, white cane sugar 6, soy oil 6, salt 2, dried yeast 1.2, and calcium propionate 0.1. The WBs were prepared by the straight dough method using the same equipment used for the GFBs. All the ingredients were weighed and mixed at 110 rpm for 7 min. The resultant doughs (300 g) were kneaded, rounded, placed into tins, and proofed at 30 °C with 85% relative humidity. The WB1 dough proofing was carried out in three steps of 52, 25, and 46 min, while the WB2 dough was proofed in one step of 2 h. Both doughs were baked at 180 °C for 14 min. After baking, the loaves were removed from their pans and cooled for 2 h at room temperature.

The GFBs and WBs were then sprayed with mold inhibitors, packed in polypropylene bags, and stored in controlled conditions (22–25 °C, 50–70% RH) for 3 days. The bread samples were analyzed 0, 24, 48, and 72 h postproduction. Four random loaves were used to monitor instrumental parameters and microbial growth at each storage time. In this way, a total of sixteen loaves were prepared for each bread type.

After the microbiological safety was confirmed, twenty-four loaves of each formulation were produced for the sensory evaluation. Six loaves were used to monitor the sensory acceptability at each storage time for each bread type.

### 2.3. Bread Evaluation

Fresh bread characterization consisted of the loaf-specific volume, crumb moisture, crumb firmness, crumb grain, and height/width ratio of the central slice, and sensory acceptability. The loaf-specific volume was determined according to AACC method 10–05.0 [23]. For the crumb analysis, the bread was sliced by hand using a specific bread slice regulator and bread knife (Imeca Indústria Metalurgica Ltd., Bauru, Brazil) to divide each loaf into 12.5 mm-thick slices. The moisture in the bread crumbs was determined in triplicate according to AACC method 44–15.02 [23]. The crumb firmness was determined according to AACC method 74–09.01 [23] using a texture analyzer (TA. XTplus, Stable Micro Systems, Surrey, UK). Texture measurements (six values) were performed on two bread slices taken from the centers of three different loaves. A 25 mm-thick slice was compressed up to 40% deformation using a 36 mm-diameter cylindrical aluminium probe at 1.7 mm/s speed. Crumb firmness was taken as the force required for compression of the bread sample by 25%.

The crumb cell structure of the digital bread slice images was assessed by adapting the method proposed by Santos et al. 2021 [24]. The slice images were acquired at 1200 dots per inch in RGB color and JPEG format with a flatbed scanner (multifunctional Epson L355 model, Epson do Brasil Indústria e Com. Ltd., São Paulo, Brazil) and processed using ImageJ software (National Institutes Health, Bethesda—EUA). The image of the center of each slice was cropped to a square of 1180 × 1180 pixels (equivalent to 25 × 25 mm^2^) and converted into a grayscale image. The image was binarized to allow the conversion into black and white. Black dots represent alveoli. An alveolar threshold of 0.0005 mm^2^ was applied. After this processing, the number of cells, the average cell size, and the percentage of cell area were determined, and the crumb grain characteristics were considered. Three slices were analyzed per treatment.

The effects of ageing on crumb moisture and firmness were monitored in accordance with [24]. To monitor microbial growth, one random loaf was used for each storage time. Samples of 200 g were collected and stored in sterile packages and frozen until the analyses were performed. The microbiological safety analysis consisted of the quantification of coliforms at 45 °C/g and *Salmonella* sp./25 g, according to bread microbiological standards established by Brazilian food legislation [25]. For the analysis of thermotolerant coliforms, the most probable number (MPN) method was used in triplicate [25]. A quantitative method, BAM (FDA), for *Salmonella* spp. was performed from selective enrichment [26], and the confirmation was performed by a biochemical test in triple sugar iron (TSI) agar, lysine iron agar (LIA), and Bactray System. In addition, the detection and quantification of molds and a yeast assay were performed by the traditional method of plating in Sabouraud agar with dextrose [27].

With respect to the panel test, all subjects gave their informed consent for inclusion before they participated in the study. The study was conducted in accordance with the Declaration of Helsinki, and the protocol was approved by the Ethics Committee of the Federal University of Sao Paulo CAAE: 44457415.5.0000.5505.

The sensory acceptability of each fresh and stored bread type was evaluated by 54 untrained panelists in one session. Bread consumers were recruited from the campus via internal announcements. They had no gluten-related diseases, had a habit of consuming bread, did not have any allergy or intolerance to any of the ingredients present in the products, and were aware that they were tasting GFBs and WBs. The panelists scored the aroma, texture, and flavor of the formulations on a 10 cm hybrid hedonic scale [28]. Samples with acceptability scores of at least 6.0 were considered acceptable. The bread slices (12.5 mm thick) were separately offered in a random sequence in polyethylene bags coded with 3-digit numbers. The evaluation was conducted in a climate-controlled (20–25 °C) sensory evaluation laboratory equipped with separate booths. The panelists rinsed their mouths with water between samples to minimize any residual effects, as previously described by Fratelli et al. (2018) [17] and Santos et al. (2018) [6].

### 2.4. Statistical Analysis

Differences in the treatment means were identified using analysis of variance (ANOVA one-way) and Tukey’s test. A simple linear correlation (Pearson correlation coefficient) was also evaluated. These data were processed using Statistica 12.0 statistical software (StatSoft Inc., Tulsa, OK, USA, 2013). Multiple factor analysis (MFA) and regression vector (RV) coefficients were applied to verify the data relationship using six tables of variables (crumb porosity and physical and sensorial parameters during storage: moisture, firmness, aroma, texture, and flavor). In the sequence, hierarchical clustering analysis (HCA) and a dendrogram chart were performed based on coordinates of the matrix of factors obtained by MFA, considering Euclidean distances, Ward’s method, automatic truncation, and the cophenetic correlation coefficient, using XLSTAT 2021.1 software (Addinsoft, New York, NY, USA, 2021).

## 3. Results and Discussion

### 3.1. Bread Quality

**Figure 1:**
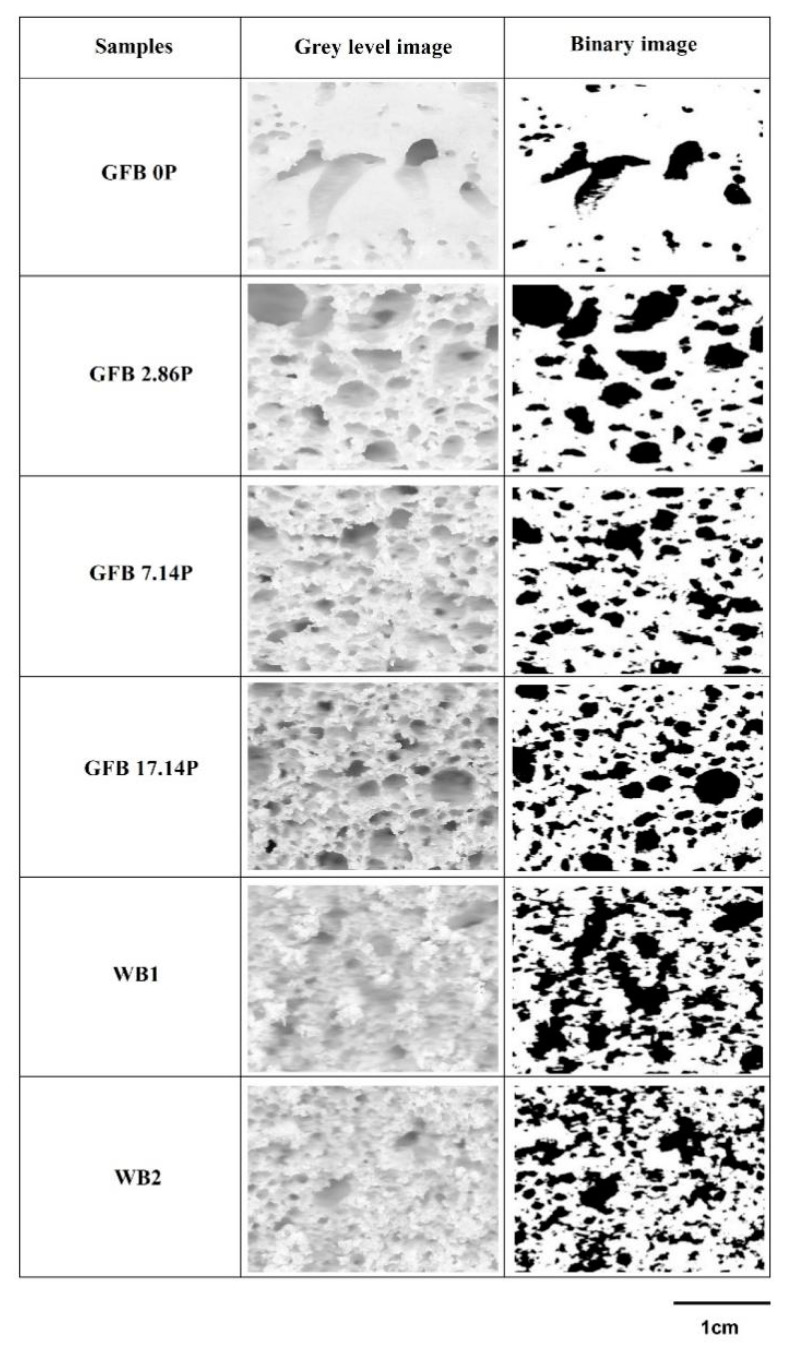
Crumb grain characteristics of gluten-free bread (GFB) formulations containing different psyllium levels (GFB0P, GFB2.86P, GFB7.14P and GFB17.14P) and the wheat counterparts (WB1 and WB2).

Table 1 shows the effect of different P levels on the physical properties and acceptability scores of fresh GFBs and WBs. Figure 1 shows the crumb cell structure of the loaves studied.

The control GFB (GFB0P) presented half the specific volume and a 9-fold higher crumb firmness than WB1 and WB2. The P addition levels with proper water levels increased the specific volume of GFB by approximately 50% and decreased the crumb firmness by approximately 70% compared to the control. The P-added GFBs (GFB2.86P, GFB7.14P, and GFB17.14P) had three-quarters of the volume and a 2.2- to 3.1-fold higher crumb firmness than the WBs.

GFB0P presented the lowest loaf-specific volume in a rectangular format (height/width ratio of 0.7) and a compact structure characterized by crumbs with a few large holes because of the cell coalescence and gas escape during breadmaking due to the more liquid batter consistency. The P-added GFB had an improved crumb grain with 2.2- to 2.7-fold more cell area than GFB0P, as shown in Figure 1, and an improved volume and format that resembled those of WB (height/width ratio varied from 0.9 to 1.3). The highest P-levels added to the GFB17.14P sample presented the highest number of cells with a rounded top. This was due to the increase in the dough consistency and strengthening of the boundaries of the expanding cells, since during heating, the characteristics of the system’s starch-hydrocolloid network intensified gas retention and improved the structural characteristics of GFB, as previously explained [20,21], effects similar to those on the WB samples.

The P-added GFB presented a higher loaf-specific volume and lower crumb firmness than the control GFB. WB1 and WB2 presented higher volumes and lower crumb firmness values than the GFB samples developed in this study. WBs presented a lower crumb moisture due to the lower water levels in their formulations compared to those of the GFBs.

During bread storage, physicochemical and microbial alterations can affect the sensory parameters of bread, especially the aroma, texture, and flavor [29]. Therefore, these parameters were chosen for the sensory acceptability evaluation of each fresh and stored bread type, with acceptability being a primordial parameter due to the direct economic relation with the consumers. Fresh GFB0P was accepted. Nevertheless, P addition improved the bread texture acceptability. No significant differences were observed in the acceptability of GFB2.86P, GFB7.14P, GFB17.14P, WB1, and WB2, and the scores ranged from 7.7 to 8.8.

Fresh P-added GFBs did not significantly differ in their physical properties and acceptability scores because the proper combinations of P and W levels defined by Fratelli et al. (2018) [17] were applied to improve GFB quality.

The results show that increasing the percentage of cell area and the loaf-specific volume decreased the crumb firmness, which improved the texture acceptability scores, and the following correlations were obtained between the loaf volume and crumb firmness (r = −0.907, P = 0.013), the loaf volume and texture acceptability (r = 0.909, P = 0.012), and the crumb firmness and texture acceptability (r = −0.941, P = 0.005). The following correlations were obtained between the percentage of cell area and loaf volume (r = 0.926, P = 0.008), crumb firmness (r = −0.975, P = 0.001), and texture acceptability (r = 0.898, P = 0.015). These data show the importance of instrumental indices of bread quality for GFB texture acceptability. In addition, a corroboration between the instrumental and sensory parameters considering the product elaborated in the present study is shown.

### 3.2. Shelf Life

**Figure 2:**
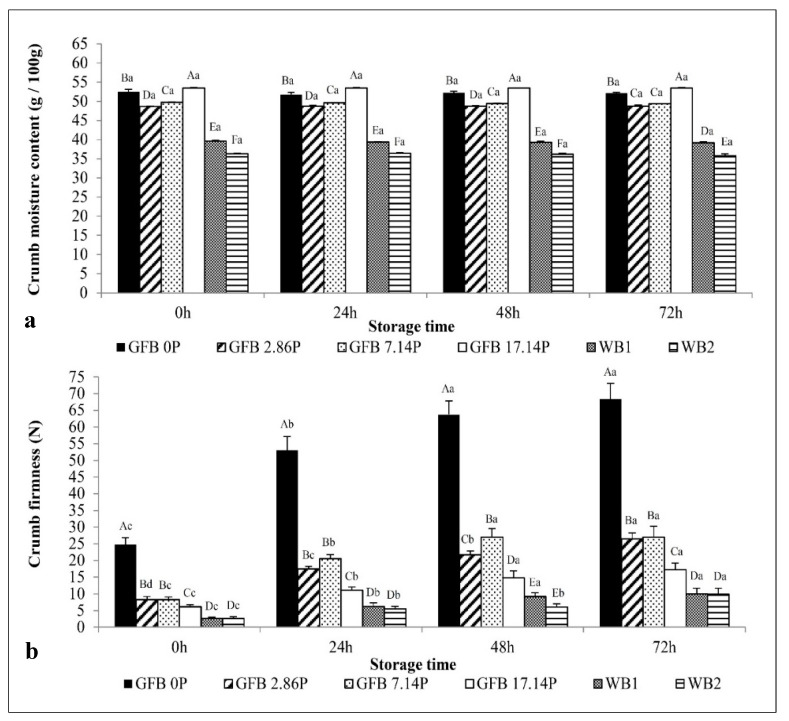
Crumb moisture (**a**) and firmness (**b**) of gluten-free bread (GFB) formulations containing different psyllium levels 0, 24, 48 and 72 h post-production and their comparison to the control and wheat counterparts. The GFBs samples GFB0P, GFB2.86P, GFB7.14P, and GFP17.14P were prepared with 0.00, 2.86, 7.14 and 17.14 psyllium (g/100g flour basis), respectively, and with adjusted water levels. WB1—standard wheat bread formulation, WB 2—adapted wheat bread formulation. Bars represent the mean values (*n* = 3 for crumb moisture and *n* = 6 for crumb firmness); error bars represent the standard deviation. Bars with different lowercase letters within the same bread type are significantly different (*p* < 0.05). Bars for a given storage time with different capital letters are significantly different (*p* < 0.05).

Figure 2 shows the evolution of crumb moisture and firmness during storage. A limited moisture decrease in bread crumb was observed during the 72 h of storage (Figure 2a) for both WB (mean crumb loss of 1.2%) and GFB (crumb loss ranges from 0.0 to 0.7%). No moisture loss during storage was observed for any of the WB and GFB loaves. The differences among the breads were due to the water addition levels in the formulations.

Crumb firming (staling) was observed during storage, as shown in Figure 2b. Furthermore, the significant differences in P-added GFBs’ and GFB0P crumb firmness demonstrated in the fresh breads were maintained and became even more evident during storage. In general, the crumb firmness values increased by nearly twofold after 24 h and nearly threefold after 72 h of storage for GFBs, while the WB crumb firmness increased by twofold after 24 h and 3.6-fold after 72 h of storage.

GFB0P exhibited firmest crumbs 0, 24, 48, and 72 h post-baking (Figure 2b). The addition of 2.86–17.14% P decreased the crumb firmness values by 65–75% compared to those of the control GFB during the storage time, without P. The best effect in delaying bread staling was noticed with a 17.14% P addition, which reduced the GFB crumb firmness by 75%. GFB0P had a crumb firmness 8-fold higher than that of the WB during the storage time, while GFB2.86P, GFB7.14P, and GFB17.14P had crumb firmness values 3- and 2-fold higher than those of WB, respectively.

Despite the higher P levels used in the present study, as well as the differences in formulations and process conditions, the results agreed with those of Cappa, Lucisano, and Mariotti (2013) [30], who reported the potential of P to decrease the GFB crumb firming ratio during 72 h of storage. A balance between the physical properties and the sensory acceptability, as well as the microbial safety, is always desired but not always achieved; fortunately, the results obtained in the present work have accomplished this equilibrium. In a recent study performed by Ziemichód, Wójcik, and Różyło (2019) [19], it was observed that after three days of storage, the addition of 5% ground seeds of Plantago psyllium, replacing rice flour in gluten-free bread, maintained the hardness and decreased the elasticity and cohesiveness of GFB crumb. All these texture aspects demonstrated lower results than the control (without P). The authors emphasize that hydrocolloid P creates a good structure that beneficially affects the texture by P addition in GFB, even though they highlight that more investigation is necessary in this area. Crumb firming during storage is expected because of various factors, such as starch retrogradation, distribution and mobility of water, and interactions among bread components [31]. The results showed that increasing the concentrations of P decreased the crumb firmness, which was mostly due to the following causes: the starch was diluted by the incorporation of P, with adjusted water levels, which increased the dietary fiber content of 2.5% in the control to 4.0–9.2% in the 2.86–17.14% P-added bread [17]. Additionally, high fiber levels may compete for water with starch, which decreases starch hydration and gelatinization, reduces the amount of retrograded starch, and decreases crumb firmness values [32]. Furthermore, the great water-binding ability of P may limit water mobility, which influences starch retrogradation and crumb firming kinetics, delaying bread staling [31,33].

In view of the staling behavior, GFB7.14P presented no advantage over the GFB2.86P formulation. Thus, considering a good cost-to-benefit ratio, GFB7.14P was not included in the investigation of the time effect on the sensory acceptability of GFB.

The results of the microbiological safety evaluation indicated that the formulations GFB0P, GFB2.86P, GFB17.14P, WB1, and WB2 are suitable for human consumption based on the current legislation. The microbiological standards of Brazilian food legislation were maintained [26]; thermotolerant coliforms and *Salmonella* sp. were absent, and no molds or yeasts were detected in the breads during storage.

**Figure 3:**
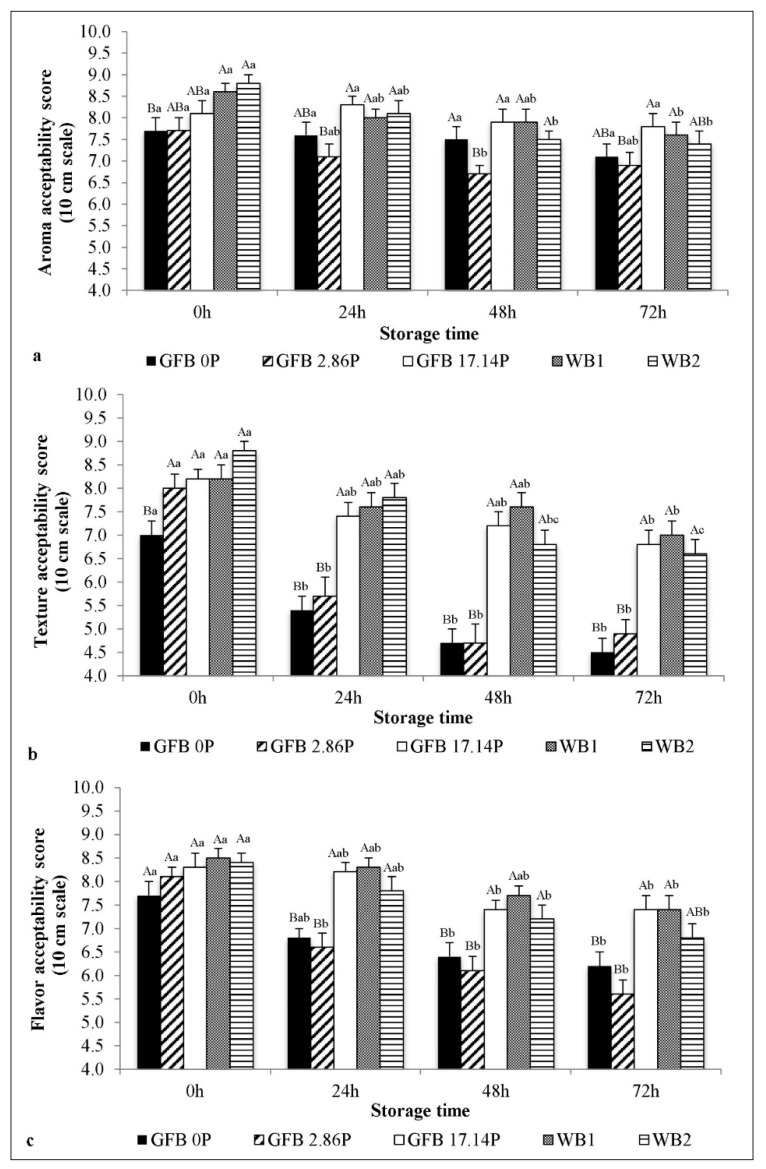
Acceptability scores for the aroma (**a**), texture (**b**) and flavor (**c**) of gluten-free bread (GFB) formulations containing different psyllium levels (GFB2.86P and GFB17.14P) 0, 24, 48 and 72 h post-production and their comparison to the control (GFB0P) and wheat counterparts (WB). The GFBs samples GFB0P, GFB2.86P, and GFP17.14P were prepared with 0.00, 2.86, and 17.14 psyllium (g/100 g flour basis), respectively, and with adjusted water levels. WB1—standard wheat bread formulation, WB 2—adapted wheat bread formulation. Bars represent the mean values (*n* = 54); error bars represent the standard deviation. Bars with different lowercase letters within the same bread type are significantly different (*p* < 0.05). Bars for a given storage time with different capital letters are significantly different (*p* < 0.05).

Figure 3 shows the acceptability scores of the breads during storage.

No significant difference in the aroma acceptability scores was observed for the GFBs during storage. However, a decrease in aroma acceptability was observed for WB1 after 72 h and for WB2 after 48 h. During the 24–72 h of storage, GFB2.86P presented the lowest aroma score, and no difference was observed among GFB0P, GFB17.14P, WB1, and WB2 during storage, which all maintained acceptable aroma scores (scores ranging from 7.1 to 7.8 when measured 72 h after baking).

GFB0P and GFB2.86P suffered a significant decrease in texture and flavor acceptability during storage. After 24 h of storage, the texture scores ranged from 4.5 to 5.7, and flavor maintained a lower score (values ranging from 5.6 to 6.8), indicating that the developed breads were not acceptable compared with the other breads during the same period. GFB17.14P suffered a significant decrease in texture and flavor acceptability after 72 h of storage, WB1 suffered a significant decrease in texture and flavor acceptability after 72 h and 48 h of storage, respectively, while for WB2, a difference in texture and flavor acceptability was found after 48 h and 72 h, respectively. GFB17.14P was well accepted during the 72 h of storage, with acceptable scores for aroma, texture, and flavor ranging from 6.8 to 8.3, which were comparable to the scores of WB1 and WB2.

The panelist scores agreed with the texture instrumental analysis, and a good correlation was found and showed that increasing crumb firmness during storage decreased the bread texture acceptability scores (r = −0.808, P = 0.000). These data show the importance of these instrumental indicators for bread quality, and crumb firmness would be a predictor of texture acceptability of both fresh and stored WB and GFB. The results show that the addition of 17.14% P to a GFB formulation successfully improves the structure, appearance, texture and acceptability, and delays bread staling. This P-enriched GFB can be considered high in fiber, since it contains 9.2% dietary fiber, and it also has a low glycemic index (GI = 50) and low glycemic load (GL = 9), as previously reported [17]. Thus, this is a very promising product, as GFBs are often recognized as lacking in those quality parameters [34,35,36].

It is important to highlight that the volunteers had no dietary restrictions, and a good acceptability evaluation by this group implies a similarity with regular products. This is very promising for consumers with gluten-related disorders because gluten-free products are often less tasty and less attractive than regular products [22].

### 3.3. Relationships between Crumb Porosity and Physical and Sensorial Properties during Storage

Table 2 shows the RV coefficients of the analyzed variables, indicating that the crumb porosity parameters presented a strong (>0.7) relationship with crumb firmness and all sensorial parameters during storage. In addition, a strong relationship between crumb firmness and sensorial texture during storage was observed (RV = 0782).

**Figure 4:**
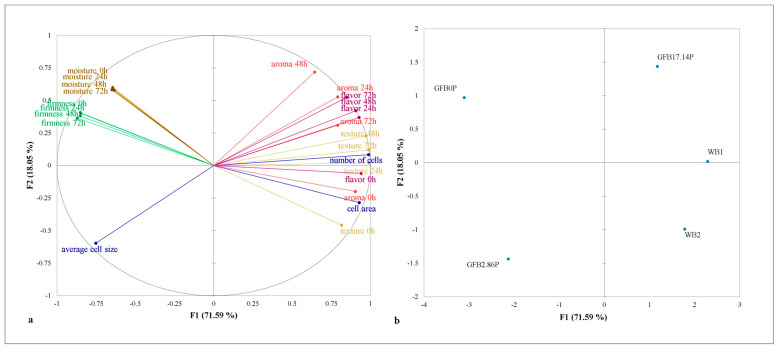
Multiple factor analysis correlating crumb porosity and the physical and sensorial properties evaluated during 72 h of storage (**a**) of gluten-free bread with (GFB2.86P and GFB17.14P) and without (GFB0P) psyllium in comparison to wheat bread (WB1 and WB2) (**b**).

Figure 4 shows the relationships between physical and sensorial properties during storage, whereby the two factors (F1 and F2) of MFA explain 89.64% of the total variation.

Factor 1 explained most of the variance (71.59%). Factor 1 was positively associated with the number of cells and cell area, as well as all the sensorial properties (except aroma at 48 h) evaluated during storage, compared to samples WB1 and WB2. Negatively, the average cell size referred to sample GFB2.86P, and the moisture and firmness during all evaluated storage periods described the sample GFB0P. Factor 2 (18.05%) discriminated only aroma at 48 h, which distinguished sample GFB17.14P.

These data indicate that the consumer scores agreed with the texture instrumental analysis, since increasing the crumb firmness during storage decreased the bread texture acceptability scores, as indicated by the inverse position of the vectors (Figure 4a) and the strong relationship observed (Table 1). These data show the importance of these instrumental indicators for bread quality, and crumb firmness would be a predictor of texture acceptability of both fresh and stored WB and GFB.

**Figure 5:**
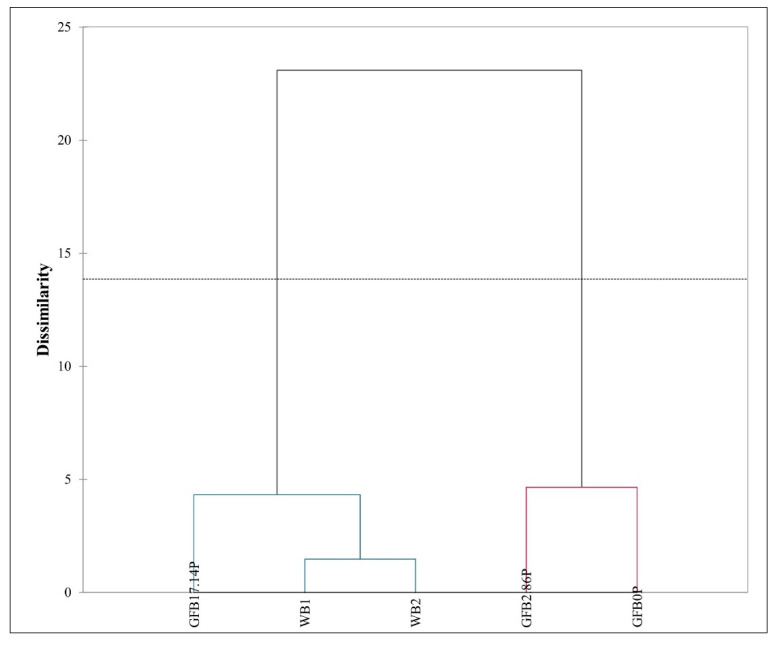
Dendogram obtained by hierarchical cluster analysis for data of gluten-free bread with (GFB2.86P and GFB17.14P) and without (GFB0P) psyllium in comparison to wheat bread (WB1 and WB2).

Figure 5 shows the existence of two groups: the first contains two products: the GFB0P and GFB2.86P samples, while the second contains three products: GFB17.1P, WB1, and WB2 samples. In addition, an adequate clustering method was observed, since it presented a high cophenetic correlation coefficient value of 0.934.

These findings indicate that the GFB17.14P sample resembled the physical and sensory properties of the WB1 and WB2 samples during 72 h of storage.

Therefore, the results show that the addition of 17.14% P in a GFB formulation successfully improves the structure, texture, acceptability, and delays bread staling, similar to traditional bread based on wheat flour.

## 4. Conclusions

The results showed that psyllium addition improved GFB structure, mouthfeel, and acceptability. No significant differences were observed in the acceptability of fresh psyllium-enriched GFBs and WB. During storage, the control GFB had a crumb firmness eightfold higher than that of WB. Psyllium addition decreased the crumb firming ratio by 65–75%. The longest delay in GFB staling was observed with the addition of 17.14% psyllium, maintaining acceptability during storage comparable to that of the WB counterparts. Therefore, our findings are useful for both GFB researchers and producers, indicating a promising alternative for obtaining healthier GFB, together with a reduction of bread staling.

## Figures and Tables

**Table 1:** Quality parameters of gluten-free bread formulations containing different psyllium levels and their comparison to the control and wheat bread counterparts.

	Gluten-Free Bread ^†^				Wheat Bread ^‡^	
	GFB0P	GFB2.86P	GFB7.14P	GFB17.14P	WB1	WB2
Specific volume (cm^3^/g) ^§^	1.41 ^c^ ± 0.02	2.15 ^b^ ± 0.12	2.06 ^b^ ± 0.04	2.08 ^b^ ± 0.05	2.73 ^a^ ± 0.10	2.84 ^a^ ± 0.08
Height/width ratio ^§^	0.67 ^c^ ± 0.01	0.90 ^d^ ± 0.01	0.91 ^d^ ± 0.04	1.00 ^c^ ± 0.02	1.29 ^a^ ± 0.01	1.11 ^b^ ± 0.01
Crumb grain ^§^						
Number of cells	43.50 ^c^ ± 0.71	72.00 ^c^ ± 2.83	132.50 ^b^ ± 14.85	160.50 ^ab^ ± 13.44	172.00 ^a^ ± 9.90	150.50 ^ab^ ± 4.95
Average size (mm^2^)	0.17 ^ab^ ± 0.02	0.22 ^a^ ± 0.04	0.13 ^b^ ± 0.01	0.12 ^b^ ± 0.01	0.14 ^b^ ± 0.02	0.14 ^ab^ ± 0.01
Cell area (%)	11.43 ^c^ ± 1.44	25.50 ^b^ ± 5.53	26.87 ^ab^ ± 1.13	30.83 ^ab^ ± 0.24	37.45 ^a^ ± 3.37	34.06 ^ab^ ± 0.64
Crumb firmness (N)	24.72 ^a^ ± 2.10	8.27 ^b^ ± 0.89	8.33 ^b^ ± 0.71	6.12 ^c^ ± 0.58	2.71 ^d^ ± 0.33	2.71 ^d^ ± 0.43
Crumb moisture (%) ^§^	52.46 ^b^ ± 0.62	48.62 ^d^ ± 0.04	49.73 ^c^ ± 0.06	53.45 ^a^ ± 0.14	39.63 ^e^ ± 0.23	36.30 ^f^ ± 0.10
Acceptability scores on a 10-cm scale ^α^						
Aroma	7.68 ^b^ ± 2.19	7.72 ^b^ ± 2.10	8.36 ^ab^ ± 1.97	8.13 ^ab^ ± 1.86	8.62 ^ab^ ± 1.32	8.75 ^a^ ± 1.54
Texture	6.96 ^b^ ± 2.38	7.96 ^ab^ ± 2.11	8.02 ^ab^ ± 1.78	8.17 ^a^ ± 2.34	8.19 ^a^ ± 1.89	8.80 ^a^ ± 1.41
Flavor	7.67 ^a^ ± 2.04	8.09 ^a^ ± 1.77	8.25 ^a^ ± 1.51	8.34 ^a^ ± 1.87	8.52 ^a^ ± 1.45	8.42 ^a^ ± 1.55

^†^ The GFB samples GFB0P, GFB2.86P, GFB7.14P, and GFB17.14P were prepared with 0.00%, 2.86%, 7.14%, and 17.14% psyllium (P) on a flour-weight basis, respectively, and with adjusted water levels. ^‡^ WB1–standard formulation, WB2–adapted formulation. Values are mean ± standard deviation ^§^ (*n* = 3), (*n* = 6), ^α^ (*n* = 54). The values followed by a different superscript in each row are significantly different (*p* < 0.05).

**Table 2:** Regression vector (RV) coefficients relating crumb porosity and the physical and sensorial properties during 72 h of storage.

	Crumb Porosity	Crumb Moisture	Crumb Firmness	Aroma	Texture	Flavor
Crumb porosity	1.000	0.303	**0.722**	**0.844**	**0.963**	**0.946**
Crumb moisture	0.303	1.000	0.369	0.313	0.376	0.143
Crumb firmness	0.722	0.369	1.000	0.324	**0.782**	0.529
Aroma	**0.844**	0.313	0.324	1.000	**0.764**	**0.886**
Texture	**0.963**	0.376	**0.782**	**0.764**	1.000	**0.883**
Flavor	**0.946**	0.143	0.529	**0.886**	**0.883**	1.000

Values in bold indicate a strong relationship (>0.7) between the six variables evaluated.

## Data Availability

Data will be made available upon request.
